# Identification of circo-like virus-Brazil genomic sequences in raw sewage from the metropolitan area of São Paulo: evidence of circulation two and three years after the first detection

**DOI:** 10.1590/0074-02760160312

**Published:** 2017-01-30

**Authors:** Silvana Beres Castrignano, Teresa Keico Nagasse-Sugahara, Patrícia Garrafa, Telma Alves Monezi, Karina Medici Barrella, Dolores Ursula Mehnert

**Affiliations:** 1 Instituto Adolfo Lutz, Centro de Virologia, Núcleo de Doenças Respiratórias, São Paulo, SP, Brasil; 2 Universidade de São Paulo, Departamento de Microbiologia, São Paulo, SP, Brasil

**Keywords:** circo-like virus-Brazil, circular DNA virus, single-stranded DNA virus, Rep gene, CRESS-DNA virus, sewage

## Abstract

**BACKGROUND:**

Two novel viruses named circo-like virus-Brazil (CLV-BR) hs1 and hs2 were previously discovered in a Brazilian human fecal sample through metagenomics. CLV-BR hs1 and hs2 possess a small circular DNA genome encoding a replication initiator protein (Rep), and the two genomes exhibit 92% nucleotide identity with each other. Phylogenetic analysis based on the Rep protein showed that CLV-BRs do not cluster with circoviruses, nanoviruses, geminiviruses or cycloviruses.

**OBJECTIVE:**

The aim of this study was to search for CLV-BR genomes in sewage and reclaimed water samples from the metropolitan area of São Paulo, Brazil, to verify whether the first detection of these viruses was an isolated finding.

**METHODS:**

Sewage and reclaimed water samples collected concomitantly during the years 2005-2006 were purified and concentrated using methodologies designed for the study of viruses. A total of 177 treated reclaimed water samples were grouped into five pools, as were 177 treated raw sewage samples. Nucleic acid extraction, polymerase chain reaction (PCR) amplification and Sanger sequencing were then performed.e

**FINDINGS CLV-BR:**

genomes were detected in two pools of sewage samples, p6 and p9. Approximately 28% and 51% of the CLV-BR genome was amplified from p6 and p9, respectively, including 76% of the Rep gene. The detected genomes are most likely related to CLV-BR hs1. Comparative analysis showed several synonymous substitutions within Rep-encoding sequences, suggesting purifying selection for this gene, as has been observed for other eukaryotic circular Rep-encoding single-stranded DNA (CRESS-DNA) viruses.

**MAIN CONCLUSION:**

The results therefore indicated that CLV-BR has continued to circulate in Brazil two and three years after first being detected.

Circoviruses, nanoviruses, and geminiviruses are eukaryotic viruses with small circular single-stranded DNA (ssDNA) genomes encoding a replication initiator protein (Rep) with specific characteristics, including three conserved rolling circle replication (RCR) motifs at structurally equivalent positions in the Rep N-terminus and superfamily 3 (SP3) helicase motifs (King et al. 2012, Rosario et al. 2012b). Pigs and numerous bird species are the hosts of circoviruses, while plants are the hosts of nano- and geminiviruses (King et al. 2012, Rosario et al. 2012b). In addition to recognised members of these three families, there is a large number of new small circular DNA genomes with similar Rep-encoding genes described in the literature that do not group phylogenetically with these viral families. Small circular Rep-encoding viral genomes have been detected in diverse samples (e.g., samples obtained from humans and other mammals, fish and insects as well as environmental samples) (Delwart & Li 2012, Rosario et al. 2012b), but their hosts are generally unknown. The genomic diversity of these new viruses is so enormous that it is only possible to construct a phylogenetic tree for them based on the Rep gene, which is the most conserved gene in the genomes of these emergent viruses. Some of these genomes have been tentatively grouped in the literature according to genomic and phylogenetic characteristics (e.g., cycloviruses, gemycircularviruses, and krikoviruses) (Li et al. 2010, Rosario et al. 2012a, Garigliany et al. 2015) [There is a tendency toward recognition of these groups by the International Committee on Taxonomy of Viruses, as happened recently with Sclerotinia sclerotiorum hypovirulence-associated DNA virus 1. This virus was previously a member of the gemycircularvirus group, but was allocated to the newly created *Genomoviridae* family, which contains a single genus, *Gemycircularvirus* (Krupovic et al. 2016)]. Nevertheless, many of these newly discovered genomes that cannot be clustered have been described as novel circovirus-like viruses (Rosario et al. 2009, 20, 2012b, Castrignano et al. 2013), and recently, with the recognition of their diversity and limited similarities to known members of the family *Circoviridae*, the term novel circular Rep-encoding ssDNA (CRESS-DNA) viruses has been coined (Rosario et al. 2012a).

We recently discovered two CRESS-DNA genomes, designated circo-like virus-Brazil hs1 and hs2 (CLV-BRs hs1 and hs2), in a human stool sample collected in 2003 (Castrignano et al. 2013). Their hosts are still unknown, as these viruses could potentially be transiting through the human gut lumen. CLV-BRs have circular unisense DNA genomes, and these viruses are likely represented by small icosahedral virus-like particles detected by electron microscopy in this sample (Castrignano et al. 2013). CLV-BR genomes exhibit four probable open reading frames (ORFs), but only ORF 3 (Rep) shows significant similarity to other sequences in GenBank. The CLV-BR hs1 and hs2 Rep sequences were found to be very similar, as were ORFs 1 and 4, but ORF 2 showed a difference of more than 150 nucleotides (nt) and 36% of the amino acid (aa) sequence. Based on the phylogenetic tree, the CLV-BR Reps are more closely related to novel CRESS-DNA Reps identified in reclaimed water and the feces of a wild rodent and a bat (Castrignano et al. 2013).

Even though other novel CRESS-DNA viruses have been detected in studies that have analysed the viral contents of feces and sewage (Blinkova et al. 2009, Li et al. 2010, Cantalupo et al. 2011, Ge et al. 2011, Ng et al. 2012, Kraberger et al. 2015), CLV-BR genomes have not been described elsewhere in the literature. In this study, we searched for CLV-BR genomes in sewage and reclaimed water samples obtained in the metropolitan area of São Paulo, Brazil, to verify whether or not the first detection of CLV-BRs was an isolated finding.

## MATERIALS AND METHODS

**Fig. 1:**
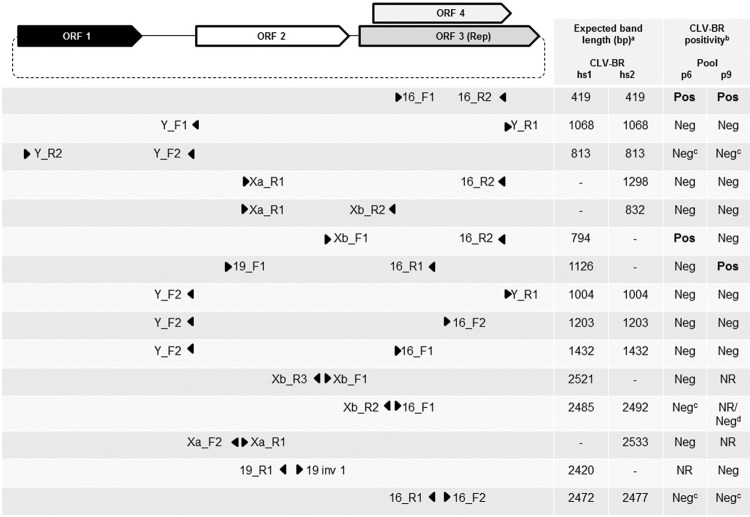
: genome organisation of circo-like virus-Brazil (CLV-BR) hs1 and hs2 and location of the primer pairs used to tentatively amplify the genomes of CLV-BRs from sewage samples p6 and p9. The dashed line indicates the circular nature of the genome. Triangles indicate the locations and orientations of the primers. Genome length: CLV-BR hs1 = 2526 nt; CLV-BR hs2 = 2533 nt. (*a*) Expected band length for CLV-BR hs1 and hs2 genomes; (*b*) CLV-BR positivity = sequencing of an expected band that resulted in a segment with similarity to CLV-BRs hs1 and hs2; (*c*) amplification with and without a pre-amplification step using the Illustra Ready-To-Go GenomiPhi V3 DNA Amplification Kit; (*d*) amplification from the product obtained after pre-amplification using the Illustra Ready-To-Go GenomiPhi V3 DNA Amplification Kit. NR = not realised; bp = base pairs.

Sewage (N = 177) and reclaimed water (N = 177) samples from a collection of the Laboratory of Human and Animal Enteric Viruses, Biomedical Sciences Institute, University of São Paulo were pooled for the assays. The original samples of sewage and reclaimed water were collected concomitantly from a sewage treatment plant located in the metropolitan area of São Paulo twice a week between January 2005 and November 2006. Viruses were concentrated from 15 L of raw sewage and 100 L of reclaimed water by filtration through Zeta Plus 60S positively charged microporous filter membranes (Cuno Inc.) followed by ultracentrifugation as previously described (Mehnert & Stewien 1993, Mehnert et al. 1997), resulting in final concentration factors of 8,000 and 50,000 times, respectively (Garrafa 2009). Cytotoxic substances and nonspecific inhibitors were eliminated by Vertrel XF (DuPont) treatment (Garrafa 2009, Queiroz et al. 2001). All reclaimed water samples were chronologically grouped into five pools (pools p1 - p5), as were the 177 treated raw sewage samples (pools p6 - p10) (Table I). Nucleic acids were extracted from these 10 pools (300 μL) using the High Pure Viral Nucleic Acid Kit (Roche Diagnostics) according to the manufacturer’s instructions. The initial polymerase chain reaction (PCR) was performed with the primer pair 16_F1/16_R2, which targets the Rep gene of CLV-BRs (Castrignano et al. 2013) (Fig. 1). Positive samples for this amplification were also subjected to amplification with other previously described primer sets (Castrignano et al. 2013) (Fig. 1). DNA polymerase (Biotools B & M Labs) was employed in all PCR assays (each with 35 cycles), and the annealing temperatures were calculated using the PrimerSelect program in the Lasergene package (DNASTAR Inc). All the first-round PCR products were reamplified (5 µL of the first-round amplification product was added to the second-round amplification mixture) because the expected bands were visible only after a second round of PCR.

Amplifications with some primer sets were repeated after a pre-amplification step for samples p6 and p9. The Illustra Ready-To-Go GenomiPhi V3 DNA Amplification Kit (GE Healthcare) was used for this purpose according to the manufacturer’s instructions, except for the duration of the reaction, which was extended to 16 hours. After this random amplification, the product was further amplified through two rounds of PCR (Fig. 1).

The expected amplicons were purified (High Pure Purification Kit, Roche Diagnostics) from the excised bands or the PCR products before sequencing using the Big Dye Terminator v3.1 Cycle Sequencing Kit (Applied Biosystems) and an ABI3130xl Genetic Analyzer (Applied Biosystems). The resulting sequences were aligned and analysed using the SeqMan and EditSeq programs of the Lasergene package and the Basic Local Alignment Search Tool (BLAST) (http://blast.ncbi.nlm.nih.gov/Blast.cgi). A maximum-likelihood phylogenetic tree (1000 bootstrap replicates) was constructed in MEGA 7 (Kumar et al. 2016) using Muscle for alignment of the aa sequences and the LG + G + I model, which was selected by MEGA 7 as the preferred model.

The partial genome sequences of CLV-BR sew-p6 and sew-p9 were deposited in GenBank under accession numbers KT369098 and KT369099, respectively.

## RESULTS

Among the five pools of sewage and five pools of reclaimed water, the target sequence of the Rep gene of CLV-BRs was found in two pools of sewage, p6 and p9, but not on any of the pools of reclaimed water (Table I). In the two positive pools, the 375-nt segment between primers 16_F1 and 16_R2 (determined with high stringency according to the SeqMan program) showed a difference of 12 nt (including the two positions where ambiguities were detected) (Table II; from nt 1942 to 2316, genome numbering according to CLV-BR hs1). A BLASTn search of this segment from p6 and p9 showed CLV-BRs hs1 and hs2 as the top hits: p6 analysis resulted in a query coverage (QC) = 100%, E value (E_v_) = 0.0, and identity (I) = 99%, and p9 analysis resulted in QC = 100%, E_v_ = 6e-175, and I = 97%.

**TABLE I:** Pools of samples and respective sampling periods

Sample	Pool	Sample numbers	Sampling period mm/dd/yy		
Reclaimed water	1	1 - 35	01/10/05	-	05/19/05
	2	36 - 70	05/23/05	-	10/03/05
	3	71 - 105	10/06/05	-	02/20/06
	4	106 - 140	02/23/06	-	06/26/06
	5	141 - 177	06/29/06	-	11/13/06
Sewage	6	1 - 35	01/10/05	-	05/19/05
	7	36 - 70	05/23/05	-	10/03/05
	8	71 - 105	10/06/05	-	02/20/06
	9	106 - 140	02/23/06	-	06/26/06
	10	141 - 177	06/29/06	-	11/13/06

**TABLE II:** Differences in the genomes of circo-like virus-Brazil (CLV-BR) detected in p6 and p9 (this study) in comparison with the previously published genome of CLV-BR hs1

CLV-BR hs1^a^	Pool 6 1599-2316		Pool 9 1027-2316	
Position^b^	≠ nucleotides	≠ amino acid	≠ nucleotides	≠ amino acid
1178	NO^c^	NO	G → A	ORF 2: D → N
1353	NO	NO	A → R (G/A)	ORF 2: N → S/N
1544	NO	NO	A → G	ORF 2: T → A
1591	NO	NO	A → G	ORF 2: --
1639	deletion (T)	(intergenic region)	deletion (T)	(intergenic region)
1796	C → T	ORF 3: -- ORF 4: T → I	C → T	ORF 3^d^: -- ORF 4: T → I
1982	T → Y (C/T)	ORF 3: -- ORF 4: M → T/M	T → C	ORF 3: -- ORF 4: M → T
2000			G → R (G/A)	ORF 3: -- ORF 4: R → K/R
2060			C → T	ORF 3: -- ORF 4: S → L
2078			G → A	ORF 3: -- ORF 4: G → E
2115			C → A	ORF 3: -- ORF 4: --
2136			A → C	ORF 3: -- ORF 4: E → D
2172			A → C	ORF 3: -- ORF 4: E → D
2204			C → T	ORF 3: -- ORF 4: P → L
2207			C → T	ORF 3: -- ORF 4: T → M
2213			T → C	ORF 3: -- ORF 4: V → A
2228	C → T	ORF 3: -- ORF 4: T → I	C → T	ORF 3: -- ORF 4: T → I
2264			C → T	ORF 3: -- ORF 4: T → M
2307			A → C	ORF 3: -- ORF 4: --

*a*: GenBank accession number = NC_023888; *b*: numbers indicate the nucleotide positions in the genome of CLV-BR hs1; *c*: NO = not obtained; *d*: ORF 3 = putative Rep.

Based on these results, a series of other PCRs were performed using primers specific for the previously described CLV-BRs (Castrignano et al. 2013). Specific amplification was achieved only with primer pair Xb_F1/16_R2 for sample p6 and primer pair 19_F1/16_R1 for sample p9 (Fig. 1). Performing random amplification prior to specific amplification did not lead to further positive amplifications (Fig. 1). The obtained consensus sequences were partial CLV-BR genomes of 717 nt and 1289 nt from p6 and p9, respectively (approximately 28% and 51% of a CLV-BR genome). BLASTn search results (with default parameters and the program optimised for highly similar sequences) showed hits only to CLV-BRs hs1 and hs2, both exhibiting E_v_ = 0.0, with a higher identity with hs1 being observed. BLASTn analysis of the 654-nt segment of the putative Rep genes of p6 and p9 showed only CLV-BRs as highly similar sequences, with CLV-BR hs1 being the top hit (QC = 100%, E_v_ = 0.0).

Table II shows the comparison between the consensus sequences obtained in p6 and p9 in relation to the CLV-BR hs1 genome.

BLASTx searches of the 654-nt/218-aa segments of the putative Rep genes from p6 and p9 mainly showed hits to CRESS-DNA virus genomes, with the top hit being the putative Rep of CLV-BR hs1, with QC = 100%, E_v_ = 7e-158 and I = 100% for both analyses. Following the exclusion of the virus taxid from this analysis, we obtained sequence homology mainly with parasite genomes, such as those of *Giardia duodenalis*, *Hymenolepis microstoma* and *Gregarina niphandrodes*, with E_v_ < 10^-5^ (Supplementary data, Table).

**Fig. 2:**
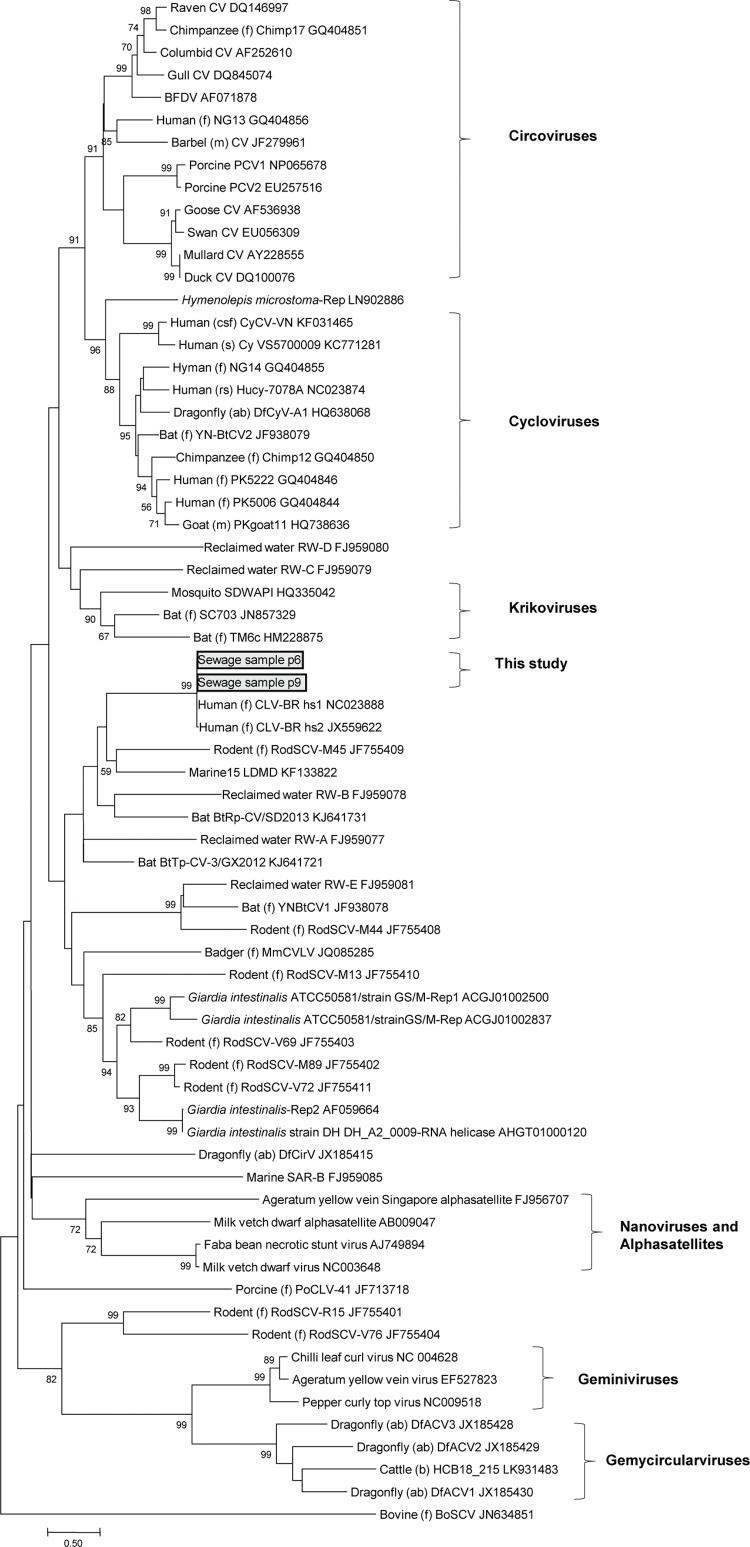
: maximum-likelihood phylogenetic analysis based on the replication initiator protein (Rep) sequences of circo-like virus-Brazil (CLV-BR), representative members of the *Circoviridae*, *Geminiviridae* and *Nanoviridae* families, alphasatellites, parasites, the proposed new genera of cycloviruses, gemycircularviruses and krikoviruses, and still unassigned novel CRESS-DNA viruses. The complete Rep sequences used as references were retrieved from GenBank. The GenBank accession number of each sequence is shown. Only bootstrap values > 50% are shown. Bar = 0.5 aa substitutions per site. Sequences identified in feces (f), meat (m), serum (s), cerebrospinal fluid (csf), abdominal samples (ab) and respiratory secretions (rs) are specified.

The phylogenetic tree based on the Rep aa sequences from viruses and the top hit parasites (Supplementary data, Table) showed that CLV-BRs do not group with circo-, nano-, gemini-, cyclo-, gemycircular-, or krikoviruses (Fig. 2). Hence, they are among the novel CRESS-DNA viruses and await taxonomic classification.

## DISCUSSION

In this study, we searched for the two recently described genomes of CRESS-DNA viruses CLV-BR hs1 and hs2 (Castrignano et al. 2013) and found CLV-BR genomic segments in two pools of sewage samples using PCR targeting the Rep gene.

Based on the fact that CLV-BRs were not detected in the pools of corresponding reclaimed water samples that were collected concomitantly, we speculated that treatment at the sewage treatment plant reduced the quantity of CLV-BRs. This reduction of virus levels is also inferred from the results obtained when adenoviruses and rotaviruses were investigated in the same samples (Garrafa 2009).

It is probable that the viruses came from human waste, considering their previous identification in human feces (Castrignano et al. 2013) and the fact that sewage consists largely of human excrement; however, other possible origins, such as animal feces (including those of domestic and agricultural animals, rodents and birds), plant material, and insects, cannot be excluded. Additionally, many of the viruses found in sewage may come from microorganisms growing within it (Cantalupo et al. 2011, Kraberger et al. 2015).

BLASTn and BLASTx analyses of the genome segments amplified from p6 and p9 showed CLV-BR hs1 as the best hit in GenBank; therefore, the discussion will be based on similarities and differences in relation to this virus genome.

We succeeded in amplifying a 654-nt segment of ORF 3 (Rep gene) from the viruses present in the two pools, representing 76% of ORF 3 of CLV-BRs. The analysis of this ORF 3 segment (654 nt/218 aa) in relation to CLV-BR hs1 (whole gene: 864 nt/287 aa) showed that while p6 exhibited a difference of 3 nt and 0 aa, p9 exhibited a difference of 14 nt and 0 aa (Table II). Thus, the motifs associated with RCR and SP3 helicase were maintained, suggesting that selective pressure has favored the function of Rep.

The putative ORF 4 (with an unknown function), which is positioned in the same genomic region as ORF 3 but in a different reading frame (Fig. 1), did not appear to have experienced strong selective pressure, as the 206-nt sequences found in p6 and p9 displayed differences of 3 and 12 aa, respectively, in relation to CLV-BR hs1 (Table II). Similar findings have been detected in other eukaryotic CRESS-DNA viruses: the mean evolutionary rate of the Rep gene of beak and feather disease virus, a member of the *Circoviridae* family, was shown to be much lower than that for the entire genome (Sarker et al. 2014), and the Reps of gemycircularviruses are more conserved than their capsid proteins based on pairwise comparisons (Rosario et al. 2012a). The 591-nt segment of ORF 2 from p9 exhibited high similarity to ORF 2 of CLV-BR hs1 (Table II), and BLASTx analysis showed the best hit with CLV-BR hs1, with QC = 99%, E_v_ = 2e-139, and I = 98%. A reliable analysis of ORF 2 of p6 was not possible due to its short length (18 nt).

The ambiguous nucleotides found at one genomic position in p6 and two genomic positions in p9 (Table II) imply that viral strains with different nucleotides at these loci were present either in a single original sample or in different samples that were pooled together. These genetic ambiguities and the above-mentioned substitutions may be due to the susceptibility of ssDNA virus genomes to mutation events (Duffy et al. 2008, Grigoras et al. 2010, Domingo-Calap & Sanjuán 2011, Sarker et al. 2014).

Primer sets that should exclusively amplify CLV-BR hs2 did not generate amplicons that could be attributed to this virus. However, some primer sets that should exclusively amplify segments of CLV-BR hs1 or both hs1 and hs2 also did not generate CLV-BR segments (Fig. 1). This result can likely be explained by the hypothesis that mutations had occurred in the annealing regions of these primers in relation to the previously described virus. This is plausible, as the ssDNA viruses display high mutation rates (Duffy et al. 2008, Grigoras et al. 2010, Domingo-Calap & Sanjuán 2011, Sarker et al. 2014) and high recombination rates (Martin et al. 2011). On the other hand, some primer pairs that should anneal to sequences recovered from p6 and p9 did not produce the expected amplification bands, as observed for the following inverse PCRs (Fig. 1): 16_F2/16_R1 for p6 (perfect annealing) and p9 (perfect annealing of 16_F2 and a 1-nt difference in the middle of 16_R1); Xb_R2/16_F1 for p6 (perfect annealing); and 19_R1/19inv1 for p9 (perfect annealing). Pre-amplification using in vitro rolling circle amplification with Phi29 polymerase also did not help us circumvent this problem.

One possibility that has to be discussed because of the unsuccessful attempt to amplify the whole virus genomes, even after a pre-amplification step with Phi29 polymerase, is that the obtained DNA sequence, instead of being encapsidated in a virion, might be inside the genome of an organism. In fact, partial genome sequences related to ssDNA viruses with characteristics of eukaryotic viruses – such as CLV-BRs – have been found to be widespread in eukaryotic genomes (Belyi et al. 2010, Liu et al. 2011), probably originating from integration relying on the endonuclease activity of their Rep (Krupovic & Forterre 2015). Many of these insertions occurred millions of years ago (Belyi et al. 2010, Liu et al. 2011), and they provide clues about infections of ancestors of present-day organisms (Belyi et al. 2010, Liu et al. 2011, Rosario et al. 2012b). The closest relatives of the Reps of CLV-BRs after exclusion of viruses were Rep sequences from some parasites (Supplementary data, Table), and the phylogenetic tree including the best hits for sequences from these parasites (Fig. 2) suggests an evolutionary relationship between CRESS-DNA viruses and some parasites and, consequently, a probable virus-host relationship, at least in ancient times (Liu et al. 2011, Delwart & Li 2012). Nonetheless, the hypothesis that the sequences obtained from p6 and p9 could be from eukaryotic hosts seems unlikely because, during the purification and concentration of the raw sewage, techniques were employed to concentrate only viruses, and the segment similarity, based on BLAST results, to genomes of parasites is much lower than to an extensive list of CRESS-DNA viruses. However, the possibility of detecting eukaryotic genomes cannot be completely excluded because we did not treat the pools with nucleases before performing DNA extraction.

A second and more probable possibility regarding the failure to amplify the whole virus genomes is that there was a very low number of copies of the viruses’ whole genomes or that degradation of their genomes occurred, either of which would make amplification by PCR and by Phi 29 polymerase difficult (Brukner et al. 2006).

The fact that only two of the five sewage pools were positive and two-round PCR was necessary to detect the virus genomes supports the latter explanation, suggesting that the occurrence of CLV-BR was probably not very frequent in the analysed sewage samples. However, it can also be speculated that the reason for the low quantity of CLV-BR viruses detected may have been associated with the applied methodology because, to concentrate free viruses and eliminate RT-PCR inhibitors (Mehnert et al. 1997, Queiroz et al. 2001), hosts that might harbor target viruses (parasites from human or animal guts, as hypothetical examples; Delwart & Li 2012) could have been excluded, and few free viruses may have been retained in the samples.

In this study, we detected CLV-BR genomes in two pools of sewage samples and described the mutations in the partially sequenced genomes in comparison with the only two CLV-BRs previously described in the literature (CLV-BRs hs1 and hs2). Although the identified genomes most likely are related to CLV-BR hs1, it is also possible that CLV-BR sew-p6 and sew-p9 show significant differences in one or more proteins in comparison with each other or the previously described CLV-BRs, as is the case for CLV-BRs hs1 and hs2. Our findings support the hypothesis that CLV-BR continued to circulate in Brazil two and three years after first being discovered.
