# Supplementary material for: Identification of circo-like virus-Brazil genomic sequences in raw sewage from the metropolitan area of São Paulo: evidence of circulation two and three years after the first detection
Source: Mem Inst Oswaldo Cruz. 2017 Jan 30;112(3):175–81. doi: 10.1590/0074-02760160312 (PMC5319365; doi:10.1590/0074-02760160312)
Supplement: Supplementary file 1 [file 0074-0276-mioc-0074-02760160312-suppl01.pdf]

TABLE

Top five BLASTx hits excluding viruses (taxid: 10239) obtained for circo-like virus-Brazil sew p6, sew p9, hs1, hs2 (on January 21, 2016)

| Query sequence submitted to BLASTx search / Results | Query coverage | E value | Identity | GenBank accession number |
|---|---|---|---|---|
| Query sequence<br>CLV-BR sew p6 - ORF 3 - nt: 63-716 | | | | |
| Putative replication-associated protein REP2 (*Giardia intestinalis*) | 96% | 2e-22 | 31% | AAF28772 |
| Replication association protein (*Hymenolepis microstoma*) | 77% | 5e-22 | 36% | CDS32196 |
| RNA helicase (*Giardia intestinalis*) | 96% | 9e-22 | 31% | ESU34896 |
| Replication-associated protein REP1, putative (*Giardia intestinalis* ATCC 50581) | 96% | 9e-20 | 35% | EES99726 |
| Replicase (*Gregarina niphandrodes*) | 96% | 9e-20 | 32% | XP_011133935 |
| Query sequence<br>CLV-BR sew p9 - ORF 3 - nt: 635-1288 | | | | |
| Putative replication-associated protein REP2 (*Giardia intestinalis*) | 96% | 2e-22 | 31% | AAF28772 |
| Replication association protein (*Hymenolepis microstoma*) | 77% | 5e-22 | 36% | CDS32196 |
| RNA helicase (*Giardia intestinalis*) | 96% | 9e-22 | 31% | ESU34896 |
| Replication-associated protein REP1, putative (*Giardia intestinalis* ATCC 50581) | 96% | 9e-20 | 35% | EES99726 |
| Replicase (*Gregarina niphandrodes*) | 96% | 9e-20 | 32% | XP_011133935 |
| Query sequence<br>CLV-BR hs1 - ORF 3 - nt: 1662-2525 | | | | |
| Replication association protein (*Hymenolepis microstoma*) | 80% | 2e-37 | 38% | CDS32196 |
| Replication-associated protein REP1, putative (*Giardia intestinalis* ATCC 50581) | 98% | 2e-31 | 33% | EES99726 |
| Rep protein, putative (*Giardia intestinalis* ATCC 50581) | 98% | 4e-31 | 33% | EES99432 |
| RNA helicase (*Giardia intestinalis*) | 90% | 5e-31 | 35% | ESU40270 |
| Putative replication-associated protein REP2 (*Giardia intestinalis*) | 89% | 1e-30 | 32% | AAF28772 |
| Query sequence<br>CLV-BR hs2 - ORF 3 - nt: 1669-2532 | | | | |
| Replication association protein (*Hymenolepis microstoma*) | 80% | 5e-38 | 38% | CDS32196 |
| Replication-associated protein REP1, putative (*Giardia intestinalis* ATCC 50581) | 98% | 5e-31 | 33% | EES99726 |
| Putative replication-associated protein REP2 (*Giardia intestinalis*) | 89% | 2e-30 | 31% | AAF28772 |
| RNA helicase (*Giardia intestinalis*) | 90% | 2e-30 | 35% | ESU40270 |
| Putative replication associated protein (*Gregarina niphandrodes*) | 85% | 2e-30 | 35% | Xp_011133866 |

ORF: open reading frame; Rep: replication initiator protein.
